# Understanding Private Sector Antimalarial Distribution Chains: A Cross-Sectional Mixed Methods Study in Six Malaria-Endemic Countries

**DOI:** 10.1371/journal.pone.0093763

**Published:** 2014-04-03

**Authors:** Benjamin Palafox, Edith Patouillard, Sarah Tougher, Catherine Goodman, Kara Hanson, Immo Kleinschmidt, Sergio Torres Rueda, Sabine Kiefer, Kathryn A. O’Connell, Cyprien Zinsou, Sochea Phok, Louis Akulayi, Ekundayo Arogundade, Peter Buyungo, Felton Mpasela, Desmond Chavasse

**Affiliations:** 1 London School of Hygiene and Tropical Medicine, London, United Kingdom; 2 Swiss Tropical and Public Health Institute, Basel, Switzerland; 3 Population Services International, Malaria and Child Survival Department, Nairobi, Kenya; 4 Association Béninoise pour le Marketing Social/Population Services International, Cotonou, Benin; 5 Population Services International Cambodia, Phnom Penh, Kingdom of Cambodia; 6 Association de Santé Familiale, Kinshasa, Democratic Republic of Congo; 7 Society for Family Health, Abuja, Nigeria; 8 Programme for Accessible Health, Communication and Education, Kampala, Uganda; 9 Society for Family Health, Lusaka, Zambia; Royal Tropical Institute, Netherlands

## Abstract

**Background:**

Private for-profit outlets are important treatment sources for malaria in most endemic countries. However, these outlets constitute only the last link in a chain of businesses that includes manufacturers, importers and wholesalers, all of which influence the availability, price and quality of antimalarials patients can access. We present evidence on the composition, characteristics and operation of these distribution chains and of the businesses that comprise them in six endemic countries (Benin, Cambodia, Democratic Republic of Congo, Nigeria, Uganda and Zambia).

**Methods and Findings:**

We conducted nationally representative surveys of antimalarial wholesalers during 2009–2010 using an innovative sampling approach that captured registered and unregistered distribution channels, complemented by in-depth interviews with a range of stakeholders. Antimalarial distribution chains were pyramidal in shape, with antimalarials passing through a maximum of 4–6 steps between manufacturer and retailer; however, most likely pass through 2–3 steps. Less efficacious non-artemisinin therapies (e.g. chloroquine) dominated weekly sales volumes among African wholesalers, while volumes for more efficacious artemisinin-based combination therapies (ACTs) were many times smaller. ACT sales predominated only in Cambodia. In all countries, consumer demand was the principal consideration when selecting products to stock. Selling prices and reputation were key considerations regarding supplier choice. Business practices varied across countries, with large differences in the proportions of wholesalers offering credit and delivery services to customers, and the types of distribution models adopted by businesses. Regulatory compliance also varied across countries, particularly with respect to licensing. The proportion of wholesalers possessing any up-to-date licence from national regulators was lowest in Benin and Nigeria, where vendors in traditional markets are important antimalarial supply sources.

**Conclusions:**

The structure and characteristics of antimalarial distribution chains vary across countries; therefore, understanding the wholesalers that comprise them should inform efforts aiming to improve access to quality treatment through the private sector.

## Introduction

In most low- and middle-income countries, the private for-profit sector is an important source of health care, with providers ranging from health facilities staffed by physicians and nurses, to pharmacies overseen by registered pharmacists, to more general types of retailers, including market stall and itinerant vendors, often with little or no formal health-related training [1]–[3]. These varied outlets constitute just the last link in a chain of businesses, which includes manufacturers, importers and wholesalers, which have an important influence on the availability, price and quality of medicines and other health-related commodities at the retail level [4].

The private sector is particularly important for the treatment of malaria [4]–[16]. Although malaria treatment is typically provided for free or highly subsidised in the public sector, in many countries private sector outlets are often the first and only source of treatment used outside the home [17], [18]. Here, consumers face a wide array of treatment choices, including artemisinin-based combination therapies (ACTs), which are the most efficacious drug regimens and the official first-line treatments in most endemic countries; non-artemisinin therapies (nATs) such as chloroquine, quinine, and sulphadoxine-pyrimethamine (SP), many of which were recommended treatments before the ACT era and the rise of parasite resistance; and artemisinin monotherapies (AMTs). Due to the risk that the irrational use of AMT poses for the development of artemisinin resistance, many countries have banned private sector sales of AMTs in oral dosage forms.

A wealth of information on private sector antimalarial supply and demand is now available from several endemic countries through ACTwatch, a five-year multi-country research project (www.actwatch.info). ACTwatch household surveys between 2008 and 2010 found that private for-profit sector patients were less likely to undergo malaria diagnostic testing and to receive ACTs, and were more likely to purchase nATs and other medicines [17]. This was corroborated by ACTwatch retail outlet surveys in 2009 and 2010. Among private for-profit outlets stocking at least one antimalarial in the six African study countries, availability of the recommended quality-assured (i.e. appear on WHO or UNICEF procurement lists) first-line ACTs ranged from 6% to 25%, while nearly 100% stocked nATs; they had a median price 5 to 23 times more expensive than the most popular nAT alternative; and their sales volumes represented less than 6% of the total antimalarial market share [19]. This is in contrast with the situation observed in Cambodia, where ACTs have been subsidised and socially marketed by Population Services International in the private sector since 2004 [20]: subsidised ACTs were available among 58% of private outlets stocking any antimalarial and accounted for nearly half of the antimalarial volumes sold through the private sector [18].

However, rigorous supply-side evidence that examines the structure and operation of the antimalarial distribution chain serving these private outlets is limited. A 2010 review identified key gaps as a lack of nationally representative studies on antimalarial distribution chains; limited information on the number and characteristics of antimalarial suppliers, their sales volumes and mark-ups, particularly on unregistered suppliers; and an absence of rigorous analysis of the factors influencing ACT availability and prices [4]. The third ACTwatch component, the supply chain study, aimed to address these gaps by conducting quantitative and qualitative studies on distribution chains for antimalarials. This paper presents novel evidence derived from nationally representative surveys and in-depth interviews on the structure and composition of antimalarial distribution chains, wholesaler business practices, and pharmaceutical sector regulation in six countries (Benin, Cambodia, the Democratic Republic of Congo (DRC), Nigeria, Uganda and Zambia).

## Methods

### Ethical Considerations

Experience from other ACTwatch studies conducted in the same locations demanding similar levels of participation found that written consent was often unacceptable to private sector respondents, who sometimes perceived providing signatures as potentially incriminating with regulatory bodies given the sensitive nature of the topics being covered. For others, insisting on written consent was found to cause confusion about the study purpose and distress participants. The use of verbal consent and the study as a whole was approved by the London School of Hygiene & Tropical Medicine Ethics Committee (No. 5466, 18 February 2009) and by ethical review boards in each country: the Comité national provisoire d’éthique pour la recherche en santé of Benin; the Cambodian National Ethics Committee for Health Research; the Comité d’Éthique de l’École de Santé Publique de l’Université de Kinshasa; the National Health Research Ethics Committee of Nigeria; the Research & Ethics Committee of the Makerere University Faculty of Medicine; and the University of Zambia Biomedical Research Ethics Committee. All potential respondents were given an information sheet in English, French or Khmer (in Cambodia), which emphasised the confidentiality of the information being collected, and if verbal consent was obtained, the interviewer attested to such by completing a signed certificate of consent.

### Country Contexts (Table 1)

The ACTwatch study countries were selected to provide a diverse range of markets from which comparisons and contrasts could be made. Consideration was given to several factors, including malaria burden, size of the population at risk, the scope and nature of pharmaceutical regulation (e.g. high vs. low; Francophone vs. Anglophone), public sector capacity and coverage, domestic antimalarial manufacturing capacity, existing antimalarial subsidy interventions and the feasibility of receiving the necessary country level authorisation to conduct the research [21].

**Table 1:** Key characteristics of malaria epidemiology, treatment policy and pharmaceutical licensing by country.

	COUNTRY					
	BENIN	CAMBODIA	DRC	NIGERIA	UGANDA	ZAMBIA
Predominant malaria parasite species [22]	*P falciparum* (100%)	*P falciparum* (63%), *P vivax* (37%)	*P falciparum* (100%)	*P falciparum* (100%)	*P falciparum* (100%)	*P falciparum* (100%)
% of population living in high transmission areas (≥1 case per 1000 population) [22]	100	44	97	100	90	100
Recommended first-line treatment for uncomplicated malaria (2010) [22]	AL	*P falciparum:* ASMQ, DHA-PP-PQ; *P vivax:* CQ, DHA-PP*	ASAQ	AL, ASAQ	AL	AL
ACT officially provided free of charge in public sector	NO	YES	YES	YES	YES	YES
Licences issued for pharmaceuticalwholesaling	YES: importer+ wholesaler	YES: importer, wholesaler+ retailer	YES: three types of wholesaler	YES: importer, two types of wholesaler	YES: wholesaler	YES: importer, wholesaler
Licences issued for pharmaceutical retailing	YES: retail pharmacy	YES: wholesaler+ retailer, depot A & B	YES: retail pharmacy, hospital pharmacy	YES: retail pharmacy	YES: retail pharmacy	YES: retail pharmacy
Licences issued for retailing of only OTC medicines	YES: rural outpost pharmacy	NO	NO	YES: PPMV	YES: drug shop	YES: drug store

P: Plasmodium; ACT: artemisinin-based combination therapy; AL: artemether-lumefantrine; ASAQ: artesunate-amodiaquine; ASMQ: artesunate-mefloquine; CQ: chloroquine; DHA-PP-PQ: dihydroartemisinin-piperaquine-primaquine; DHA-PP: dihydroartemisinin-piperaquine; OTC: over-the-counter; PPMV: Proprietary Patent Medicine Vendors. * As part of the programme to contain the spread of artemisinin resistance, Cambodia’s treatment guidelines until early-2011 recommended the use of DHA-PP in the highest risk areas (combined with PQ where safe use has been demonstrated) and ASMQ everywhere else to treat P falciparum malaria, and DHA-PP for the treatment of P vivax malaria since 2011 (CQ was used previously). Since early-2011, Cambodia’s treatment guidelines have recommended the use of DHA-PP (combined with PQ where safe use has been demonstrated) for both P falciparum and P vivax malaria. [22].

In all the African study countries *Plasmodium falciparum* (*Pf*) is the dominant malaria species, and over 90% of the population live in areas of high transmission. In Cambodia, 44% of the population lives in high transmission areas and infections with *Plasmodium vivax* (*Pv*) account for over a third of malaria cases [22]. At the time of data collection in 2009–2010, all study countries had already adopted ACT as the first-line treatment for uncomplicated malaria, and banned the distribution of AMT in oral dosage forms. In addition, national treatment policies were changed to recommend that patients with suspected malaria undergo a diagnostic test using either microscopy or rapid diagnostic tests (RDT). Although, treatment based on a clinical diagnosis was still recommended for suspected cases in children less than five years of age in Benin, Nigeria and Uganda; and in the DRC testing was only prescribed in cases of treatment failure or complicated malaria.

Licensing regulations for private businesses vary somewhat across countries. In the African study countries, there are separate licences for operating wholesale and retail pharmacies, both of which permit the sale of all registered pharmaceutical products and require businesses to be staffed by a supervising registered pharmacist. In Cambodia, there are three types of pharmaceutical business licenses permitting the sale of all registered pharmaceuticals: ‘pharmacies’ sell on both a wholesale and retail basis, and are managed by a registered pharmacist; retail-only ‘depot A’ and ‘depot B’ businesses are managed by an assistant pharmacist or a retired public health staff member with a minimum qualification of nurse or midwife, respectively [23]. In all countries except Cambodia and the DRC, licences are also issued to operate smaller retail businesses that are permitted to sell a limited range of over-the-counter (OTC) medicines. Often known as drug shops (or Proprietary Patent Medicine Vendors, PPMVs, in Nigeria), these retailers are not operated by registered pharmacists.

### Quantitative Methods

Sampling for the ACTwatch supply chain study drew on the nationally representative ACTwatch surveys of antimalarial retail outlets [18], [19]. During these surveys, all eligible outlets were asked to provide contact information for their top two antimalarial suppliers; and each instance a supplier was identified in this way was termed a ‘mention’. Supplier mentions collected from private sector outlets and any mentions from public sector outlets identifying private sector suppliers were used to create the sampling frame for the first level of wholesalers, called ‘terminal wholesaler’ (i.e. wholesalers supplying outlets). In smaller countries, all supplier mentions from the ACTwatch outlet survey sample were used to create the terminal-level sampling frame, while in larger countries supplier mentions from only a sub-sample of outlets were used (Table 2). We attempted to interview all wholesalers identified on the sampling frame. This process was repeated with all terminal wholesalers interviewed to identify businesses operating one level higher in the distribution chain (i.e. ‘intermediate-1 wholesalers’), and yet again (i.e. ‘intermediate-2 wholesalers, etc.) until only importers or manufacturers were identified as supply sources. At this point, the top of the distribution chain was deemed to have been reached. This ‘bottom-up’ sampling approach facilitated the identification of all types of wholesale suppliers, including those that might otherwise be excluded because they do not possess the appropriate licence from the regulatory authority (e.g. unlicensed businesses, licensed retailers that wholesale).

**Table 2:** Sample breakdown - number of wholesalers identified and interviewed, and antimalarial products audited.

	COUNTRY					
	BENIN	CAMBODIA	DRC	NIGERIA	UGANDA	ZAMBIA
Dates of data collection	4–29 Jun 2009	21 Aug–1 Nov 2009	11 Jan–10 Mar 2010	18 Jul–8 Sep 2009	13 Feb–6 Apr 2009	28 Feb–6 May 2009
Number of ACTwatch Outlet Survey clusters used to form terminal wholesaler sampling frame (over the total number of clusters)	19/19	20/38	32/76	20/76	38/38	38/38
Number of wholesalers identified through supplier mentions for thequantitative survey	228	141	179	213	170	57
- Number of refusals	10	5	0	27	4	1
- Number of duplicates	0	18	18	8	28	0
- Number not eligible	1	9	1	5	1	9
- Number not found	10	10	11	19	2	1
- Number not interviewed for other reasons	3	4	10	14	6	2
Number of quantitative wholesaler interviews conducted	204*	95	139	140	129	44
Number of antimalarials audited	1529	230	1962	2600	1326	288
Number of qualitative in-depth interviews conducted	33	43	36	39	45	42

*Results from Benin are weighted to adjust for over- or under-sampling that may have occurred due to the high number of wholesalers operating within traditional markets.

In Benin and Nigeria, traditional markets that are common throughout West Africa are important sources of antimalarial wholesaling, posing several challenges to sampling and data collection. Because some market-based respondents were reluctant to be interviewed due to political and regulatory sensitivities surrounding medicine-selling in markets, attempts were made to replace any refusals with respondents from similar businesses operating within the same market. Also, because markets do not all operate every day of the week, it was not possible to survey market wholesalers in each of the identified market towns, in which case wholesalers were sampled from markets in nearby towns to represent them. While market-based supply sources were typically mentioned only by retailers in Nigeria, both retailers and wholesalers in Benin commonly said they purchased antimalarials from markets sources, often without naming a specific vendor and giving only the market name. As such, it was difficult to ascertain which and how many wholesalers needed to be sampled in each of the identified markets in Benin, potentially leading to over- or under-sampling of market wholesalers. In the absence of a sampling frame for unlicensed market wholesalers in Benin, we used the frequency of market supplier mentions relative to the total number of supplier mentions from the quantitative survey to approximate the distribution of market-based wholesalers across all identified markets in Benin and also to calculate pseudo-probability weights, which were used to adjust results to account for over- or under-sampling of market wholesalers and applied in Stata v.11 and v.12 [24], [25] using a variety of commands that support the *aweight* and *pweight* options (see supporting information Text S1 for details on sampling in markets and the calculation of weighted summary measures). Summary measures for Nigeria were not weighted because, unlike in Benin, most market-based supplier mentions named specific businesses and were gathered predominantly from retailers. As the number of market-based wholesalers to be interviewed in various locations in Nigeria was much clearer, the risk of over- and under-sampling was minimal. In addition, summary measures for the remaining countries were not weighted because all wholesalers could be identified from the supplier mention information collected from respondents.

All identified wholesalers that could be located were screened for eligibility, with the full questionnaire administered if they had either an antimalarial or RDT in stock at the time of interview, or they reported having stocked either antimalarials or RDTs in the three months prior to interview. In each business, trained local interviewers sought to speak with the person most knowledgeable about their antimalarial wholesale business.

Data collection tools were piloted and adapted for each country setting. A structured questionnaire was used to collect data on each wholesale business’s characteristics and operations and on the wholesalers’ top two supply sources for antimalarials. Inventory sheets were used to record each antimalarial stocked, including brand, generic name, strength, package type and size, recall of volumes sold over the previous week, recall of last purchase value, and selling and purchase prices. Data collection for the supply chain study in each country was timed to follow shortly after the ACTwatch outlet survey and to coincide as much as possible with periods of peak malaria transmission (Table 2).

All data were double entered using EpiData v.3.1 and analysed with Stata v.11 and v.12. Descriptive characteristics of wholesalers are presented as percentages with 95% confidence intervals or medians with inter-quartile range. Sales volumes are presented in terms of adult equivalent treatment doses (AETD), a standardised unit which allows meaningful comparisons between antimalarials with different treatment regimens [19], [21], [26]. Where respondents could not recall or refused to provide sales volume information, volumes were imputed using multiple imputation methods based on the *mi impute pmm* command in Stata. The supporting information in Text S2 and footnotes to Table 3 present additional details on the volumes analysis.

**Table 3:** Median number of AETDs of antimalarials sold during the week preceding the survey.

			COUNTRY^2^					
ANTIMALARIAL TYPE			BENIN	CAMBODIA	DRC	NIGERIA	UGANDA	ZAMBIA
Formulation^1^			N = 201	N = 93	N = 137	N = 136	N = 127	N = 40
**ACT**	All	**Median**	**0.0**	**2.0**	**68.5**	**137.9**	**22.0**	**35.7**
		IQR	0.0–45.0	0.0–10.0	7.5–327.5	12.8–794.0	4.8–94.4	0.8–176.8
	Tablet	**Median**	**0.0**	**2.0**	**59.7**	**120.0**	**21.9**	**31.0**
		IQR	0.0–35.0	0.0–10.0	7.4–287.9	11.8–730.4	4.0–86.7	0.0–96.3
	Oral liquid	**Median**	**0.0**	**0.0**	**1.9**	**0.0**	**0.0**	**0.0**
		IQR	0.0–0.0	0.0–0.0	0.0–29.3	0.0–26.3	0.0–0.0	0.0–3.8
**AMT**	All	**Median**	**0.0**	**0.0**	**8.3**	**42.0**	**15.0**	**0.0**
		IQR	0.0–0.0	0.0–0.0	0.0–83.7	3.8–272.1	2.3–34.8	0.0–9.2
	Tablet	**Median**	**0.0**	**0.0**	**0.0**	**28.1**	**7.7**	**0.0**
		IQR	0.0–0.0	0.0–0.0	0.0–10.1	0.0–155.3	0.0–18.8	0.0–0.0
	Oral liquid	**Median**	**0.0**	**0.0**	**0.0**	**1.0**	**0.0**	**0.0**
		IQR	0.0–0.0	0.0–0.0	0.0–4.3	0.0–17.3	0.0–0.0	0.0–0.0
	Injectable	**Median**	**0.0**	**0.0**	**0.0**	**0.0**	**0.0**	**0.0**
		IQR	0.0–0.0	0.0–0.0	0.0–15.1	0.0–2.6	0.0–9.5	0.0–3.9
**nAT**	All	**Median**	**222.1**	**0.0**	**327.8**	**562.9**	**304.9**	**320.9**
		IQR	21.3–1104.1	0.0–0.0	65.3–1519.0	163.6–2006.6	52.1–1523.7	13.3–1260.4
	Tablet	**Median**	**141.9**	**0.0**	**226.2**	**392.3**	**203.8**	**301.6**
		IQR	9.5–809.5	0.0–0.0	24.5–1167.7	90.4–1649.2	10.3–1053.9	0.0–1200.0
	Oral liquid	**Median**	**0.0**	**0.0**	**6.6**	**26.8**	**28.8**	**0.0**
		IQR	0.0–15.3	0.0–0.0	0.0–70.6	0.0–101.8	3.6–78.8	0.0–3.4
	Injectable	**Median**	**0.0**	**0.0**	**0.0**	**0.0**	**0.0**	**0.0**
		IQR	0.0–0.0	0.0–0.0	0.0–13.9	0.0–4.9	0.0–19.0	0.0–0.0

N: number of wholesalers included in the sales volume analysis; ACT: artemisinin-based combination therapy; AMT: artemisinin monotherapy; nAT: non-artemisinin therapy; IQR: inter-quartile range. 1 The values for median number of AETDs sold reported for ‘all’ formulations include all dosage forms (tablets, suppositories, oral liquids, injectables and granules); however because so few wholesalers stocked suppositories or granules, and so few of these product types were observed during the audit, these dosage forms have been excluded from the tables here. 2 Notes on imputation: The number of wholesalers included in N whose sales volumes were set to zero as they did not stock antimalarials at the time of the survey but did at some point during the 3 months preceding the survey was 2 in Benin, 5 in Cambodia, 2 in the DRC, 2 in Nigeria, 1 in Uganda and 0 in Zambia. The number of wholesalers identified during the study for whom sales volumes were excluded or set to missing because they did not meet inclusion criteria or for various other reasons (see Table 2) was 17 in Benin, 9 in Cambodia, 24 in the DRC, 68 in Nigeria, 14 in Uganda and 17 in Zambia. The percentage of audited antimalarial products that had missing sales volumes data which was imputed using the mi impute pmm command in Stata was 23.7% in Benin, 3.9% in Cambodia, 9.0% in the DRC, 35.7% in Nigeria, 3.4% in Uganda and 13.3% in Zambia.

### Qualitative Methods

We conducted in-depth interviews with a subset of antimalarial wholesalers and retailers to further explore a range of topics related to the structure and composition of the market and distribution chain; provider conduct (e.g. transport of drugs, credit, source and cost of capital, marketing techniques, how stocking and supplier choices are made); and perceptions of the appropriateness of regulations and the enforcement capacity of authorities. Using the businesses participating in the quantitative survey as a sampling frame, interviewees were purposively selected at various levels of the distribution chain from manufacturers and importers down to retailers, and across various settings (i.e. urban vs. rural location; accessible vs. remote market) to capture a diverse range of experiences, practices and opinions [27]. Similar interviews were also conducted with key public and private sector stakeholders situated at the top of the distribution chain identified through a review of relevant documents and consultation with actors familiar with the country’s antimalarial market. A member of the research team from the London School of Hygiene & Tropical Medicine conducted the interviews using a semi-structured interview guide, which was informed by existing literature and the study’s aims and objectives. Given the sensitivity of some topics discussed during these interviews, detailed notes of discussions were taken by a trained local research assistant, rather than having them recorded and transcribed. As such, narrative examples rather than verbatim quotes are used to illustrate or explain themes.

Using a thematic analysis approach [28], all interview notes were read to identify the main themes or experiences. An initial coding structure of the main themes was developed based on the research questions and existing literature, which was then applied by one team member to interview notes and revised as analysis proceeded by adding additional codes and sub-codes to capture as many nuances in the data as possible. To ensure consistency across countries, co-coding exercises were conducted at the beginning of the coding process where pairs of researchers independently coded a minimum of 5 interview transcripts and then compared coding. Any discrepancies were discussed and agreed between coders [28]. Data from related themes were grouped together and summarised by noting the frequency and range of terms, concepts, practices or experiences described by respondents. Differences across distribution chain levels and countries were noted. Coding and thematic analysis was conducted using NVivo 8 software. Information from these in-depth interviews was supplemented with a review of relevant documents on antimalarial regulation and policy.

## Results

### Overview of the Sample

Using the ‘bottom-up’ sampling method described above, we identified 988 antimalarial wholesale sources operating at various distribution chain levels. Of these, 26 were not eligible to participate because they did not have antimalarials in stock at any point during the three month period prior to the survey, 47 refused, 125 were later found to be duplicate mentions or could not be found, and a further 39 were not interviewed for other reasons (e.g. a suitable respondent was not available after three attempts, the business had closed down or moved to an unknown location). Across the six study countries, we conducted a total of 751 quantitative wholesaler interviews, audited 7935 antimalarial products, and conducted 238 in-depth interviews (Table 2). The first three sections below present findings on the overall structure and other characteristics of the antimalarial distribution chain in each country, followed by two sections describing wholesaler practices related to product and supplier choice, and the final section examines regulation of the wholesale pharmaceutical sector.

### Distribution Chain Structure and Supplier Interactions

As expected, the antimalarial distributions chains in each country had a pyramidal shape with many wholesalers supplying retailers at the bottom of the chain and fewer businesses importing or purchasing antimalarials directly from domestic manufacturers. Wholesalers performed distinct functions in the chain: those selling directly to retailers (i.e. terminal wholesalers), those selling to other wholesalers (e.g. intermediate-1 wholesalers supplying terminal wholesalers), and those who imported and/or manufactured antimalarials (i.e. top-level/primary wholesalers). The proportion of wholesalers that identified a manufacturer as one of their top two antimalarial supply sources ranged from 5% in Cambodia to 56% in Nigeria (Table 4).

**Table 4:** Wholesaler characteristics and business practices.

		COUNTRY					
WHOLESALER CHARACTERISTICS		BENIN	CAMBODIA	DRC	NIGERIA	UGANDA	ZAMBIA
Years in operation	**Median**	**8**	**10**	**7**	**8**	**8**	**9**
	IQR	4–15	6–16	4–13	5–17	3–11	7–16
	(N)	(182)	(89)	(133)	(129)	(123)	(42)
Number of people working at outlet	**Median**	**3**	**2**	**9**	**5**	**6**	**8**
	IQR	2–4	2–3	4–12	3–8	4–9	5–15
	(N)	(199)	(94)	(138)	(134)	(128)	(43)
Buy directly from antimalarial manufacturers (as one of two top antimalarial suppliers)	**%**	**10.9**	**5.3**	**28.7**	**55.8**	**10.2**	**41.5**
	95% CI	3.7–18.0	0.6–9.8	21.0–36.4	47.1–64.5	4.9–15.6	25.7–57.2
	(N)	(190)	(95)	(136)	(129)	(127)	(41)
Deliver antimalarials to customers	**%**	**7.6**	**23.7**	**23.0**	**32.6**	**32.0**	**67.4**
	95% CI	2.5–12.7	14.8–32.5	15.9–30.1	24.7–40.5	23.8–40.2	52.9–82.0
	(N)	(202)	(93)	(139)	(138)	(128)	(43)
Provided credit to customers in the past 3 months	**%**	**55.3**	**39.6**	**35.5**	**77.9**	**75.0**	**62.8**
	95% CI	45.1–65.5	29.3–49.8	27.4–43.6	70.9–85.0	67.4–82.6	47.7–77.8
	(N)	(201)	(91)	(138)	(136)	(128)	(43)
Most common terms of credit offered in the past 3 months(number of days)	**Median**	**15**	**30**	**30**	**14**	**30**	**30**
	IQR	10–30	3–90	10–30	7–30	14–30	30–30
	(N)	(105)	(36)	(45)	(102)	(93)	(25)

IQR: inter-quartile range; CI: confidence interval; N: number of wholesalers contributing to calculation of indicator.

**Figure 1:**
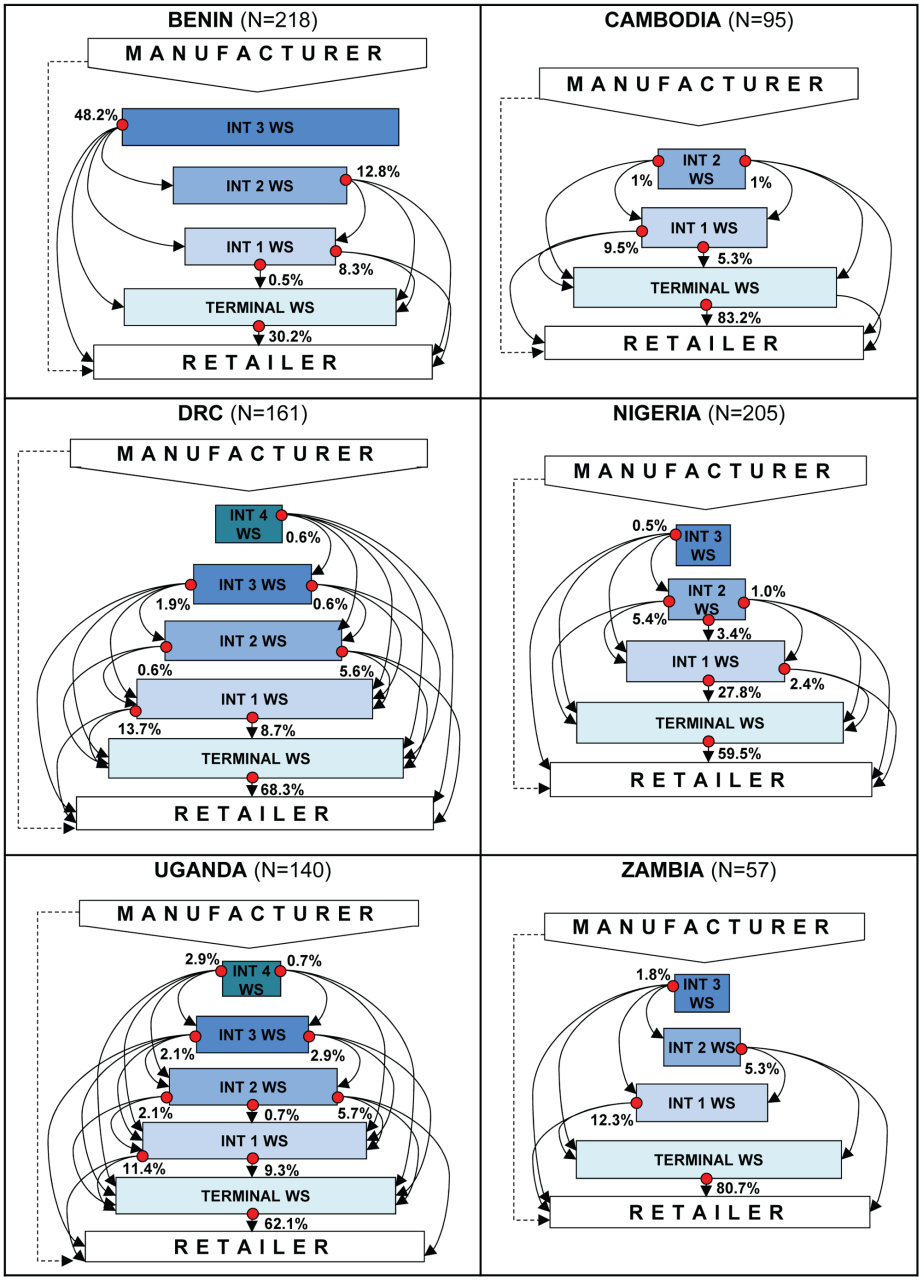
Representation of the antimalarial distribution chain illustrating the types of supplier interactions documented by country. N: number of wholesalers with documented supplier interactions; WS: wholesaler; INT: intermediate. The shaded boxes represent the different levels of the distribution chain at which wholesalers operate, and the size of each box gives an impression of the proportion of wholesalers operating at each level. The dots represent mutually exclusive groups of wholesalers that are defined by the specific levels each wholesaler group serves. This is reflected in the array of arrows emanating from each dot, which illustrates that some wholesaler groups supply several distribution chain levels and others supply only one level. The percentages attached to each dot give the relative size of each wholesaler group. The dashed line from manufacturer to retailer indicates that while some retailers purchased antimalarials directly from manufacturers, it was an uncommon practice. Note that these schematics were constructed using information about the top two antimalarial supply sources mentioned by respondents, and therefore reflect the most important supplier interactions occurring within the antimalarial distribution chain, rather than all possible interactions or the volumes of antimalarials flowing through the chain.

While many wholesalers performed a single function within the distribution chain (e.g. selling only to retailers), others performed multiple functions, operating simultaneously on several levels within the chain (e.g. sold directly to retailers and other wholesalers). Figure 1 conveys the complexity of these supplier relationships within the private sector distribution chain by illustrating the antimalarial supplier-customer interactions documented as part of the ‘bottom-up’ sampling approach in each country. In the figure, each dot represents a group of wholesalers that supply businesses operating at other levels the distribution chain (each of these levels is represented by a labelled box). The array of arrows emanating from each dot shows the specific levels each wholesaler group serves, with some groups of wholesalers serving multiple levels and others serving only one level. For example, more than 80% of wholesalers in all countries except Nigeria sold antimalarials directly to retailers; however the proportion of wholesalers supplying retailers only ranged considerably across countries, from 30% of wholesalers in Benin to 81% in Zambia. In contrast, the proportion of wholesalers only supplying other wholesalers was highest in Nigeria (37%) and lowest in Zambia (0%). These schematics also suggest that the maximum number of steps antimalarials could pass through between manufacturer and retailer ranges from 4 (Cambodia) to 6 (DRC and Uganda); however, the supplier dynamics and complexities described above indicate that most private sector antimalarials in the study countries were likely to pass through only 2 to 3 steps from production to retail.

Intersectoral transactions where private sector wholesalers identified public or non-governmental medicine distributors as important sources of antimalarials were observed in most countries. However, these types of transactions were not common, apart from in Benin where the national procurement agent, *Centrale d’Achat des Médicaments Essentiels et consommables médicaux* (CAME), was mentioned by 9.2% of all wholesalers interviewed as one of their top two antimalarial supply sources. Such private sector supply transactions are within the remit of CAME.

There was considerable variation in the role of domestic manufacturers of antimalarials across the countries. Some had vibrant domestic pharmaceutical sectors: there were over 100 licensed antimalarial manufacturers in Nigeria in 2008, 22 in the DRC, and 11 in Uganda, producing a diverse array of antimalarials, including ACTs, though none of these ACTs were prequalified by the World Health Organization at the time of data collection. In contrast, there were fewer than 4 manufacturers in each of the remaining study countries, producing only a handful of antimalarial products, none of which were ACTs.

### Traditional Markets

In Benin, Cambodia and Nigeria, traditional “open air” markets located in commercial cities and towns were important sources of antimalarial supplies for private sector retailers and wholesalers. For example, 67% of all supplier mentions gathered from retailers in Benin, and 55% in Nigeria referred to wholesalers based in traditional markets. Wholesalers in Benin and Nigeria also reported market-based businesses as important supply sources, but this was more prominent in Benin where 60% of all wholesalers surveyed counted at least one market-based supplier among their top two antimalarial supply sources.

There were some differences across countries in these markets and the antimalarial wholesalers operating within them, particularly when comparing Nigeria and Benin. In both countries, antimalarial wholesaling took place in the key traditional markets located in national and regional commercial hubs (Lagos, Onitsha and Kano in Nigeria; Cotonou and Porto Novo in Benin), where wholesalers tended to operate out of permanent buildings within the market, but also often maintained off-site warehouse facilities. Antimalarial wholesaling was also observed to take place in many smaller traditional markets in smaller city and town centres. In Benin, these wholesale businesses were sometimes observed to operate out of less permanent structures (e.g. market stalls) and traded often only on specific market days, while in Nigeria comparable wholesalers tended to operate out of permanent buildings. This is because in Nigeria most of these market-based wholesalers were operating as drug stores/PPMVs, while in Benin all market-based wholesalers were completely unlicensed (see section on regulation below).

### Wholesaler Sales Volumes

During in-depth interviews with wholesalers in African countries, antimalarials were cited as top selling products out of all types of medicines for many wholesalers and were key revenue generators. Antimalarial sales volumes among African wholesalers were overwhelmingly dominated by the sale of nATs, such as chloroquine, quinine and SP, ranging from a median of 222 AETDs sold during the week preceding the survey in Benin to 563 AETDs in Nigeria. Sales volumes of nATs were many times larger than those for ACTs, which ranged from a weekly median of 0 AETDs in Benin to 138 AETDs in Nigeria. This is in stark contrast with the very low wholesaler antimalarial volumes in Cambodia, where antimalarials were a much smaller part of overall turnover, and the median weekly sales volume was 2 AETDs for ACTs and 0 AETDs for nATs. Non-negligible weekly median sales of AMTs were only observed among wholesalers in the DRC (8 AETDs), Nigeria (42 AETDs) and Uganda (15 AETDs), most of which were sold in banned oral dosage forms (Table 3). Based on recorded sales volumes, nAT products were the top-selling antimalarial for more than 60% of wholesalers in African countries, while the recommended first-line ACT was the top-selling antimalarial among 57% of Cambodian wholesalers.

### Choice of Product

Insight gained about wholesaler product selection during qualitative interviews helps to explain some of this disparity in ACT and nAT sales volumes. In all countries, consumer demand was the principal consideration for wholesalers when selecting products to stock as this affected demand at all levels of the distribution chain. Wholesalers believed that consumer demand, in turn, was affected by several key factors, including affordability in the African study countries and popular perceptions of a medicine’s quality and efficacy in all countries. In all countries but Zambia, wholesalers’ concerns about quality and efficacy were coupled with concerns about the degree of counterfeit and substandard medicines perceived to be circulating within the national market.

In Cambodia, popular opinion about the recommended first-line ACT product’s efficacy was said to be positively affected by ongoing social marketing campaigns; however, perceived side effects of the mefloquine component of ASMQ were also believed to limit consumer demand. This concern about side effects was also voiced by many wholesalers in the DRC regarding the amodiaquine component of ASAQ. In Uganda and Zambia, some wholesalers decided not to stock the recommended first-line treatment, particularly the brand Coartem, because it was being offered free of charge in government facilities, reducing consumer demand for these products in the private sector. However, the frequency of Coartem stock outs in the Zambian public sector supply chain led some private wholesalers supplying public sector customers (e.g. district hospitals) to stock this product. Poor or irregular availability of first-line drugs among suppliers also affected wholesaler stocking decisions. Factors affecting availability reported in Nigeria included shortages of the active pharmaceutical ingredients to manufacture ACTs, delays in importation (e.g. for clearance of consignments), exchange rate volatility, fuel shortages, supplier difficulty in maintaining delivery vehicles, and issues with the couriers contracted to deliver orders.

In all countries apart from Benin, stocking decisions at higher levels of the distribution chain were constrained by exclusive distribution rights granted by foreign manufacturers to selected importers, particularly for ACTs. To qualify for such rights, importers are often required to register the foreign manufacturer’s products with the regulatory authorities for introduction to the market (e.g. conduct product analyses and obtain certificates of compliance) and also house local sales and/or medical representatives (i.e. embedded sales force) who actively promote the manufacturer’s products to prescribers, pharmacists and pharmaceutical businesses.

### Choice of Supplier

In this section, we present results related to what businesses consider when selecting suppliers, and supplier strategies for attracting customers. Among the factors affecting choice of antimalarial supplier cited by wholesalers, selling price was a key consideration in most countries. Other common factors related to a supplier’s reputation for being knowledgeable about medicines or for selling quality medicines; whether a supplier reliably stocked a sufficient range of products to fill entire orders; and whether suppliers offered promotions or discounts. In all countries, wholesalers provided discounts for orders of larger volume or value, and wholesalers in Benin also described giving discounts to customers paying in cash rather than with credit. A number of respondents in the DRC described giving gifts to customers at the end of the year such as pens, calendars, free samples, or appliances, typically related to the total annual value of a customer’s purchase.

Offering credit was another key means by which wholesalers attracted customers; however, the availability of supplier credit varied considerably across the study countries, ranging from just over a third of wholesalers in Cambodia and the DRC, to about half in Benin, and more than two-thirds in Nigeria, Uganda and Zambia (Table 4). Median credit terms ranged from 2 to 4 weeks across all countries. Credit facilities were often only extended to long-term customers and the terms could depend on factors such as past repayment history. As such, it was uncommon for wholesalers to rely entirely on credit to finance their stocking, with most using a combination of cash and credit, or cash alone.

Convenience was also a common consideration when choosing a supplier, related both to proximity and whether or not a supplier offered delivery services for orders. The proportion of wholesalers who reported delivering orders to customers varied widely, from a high of around two-thirds of wholesalers in Zambia, to less than a third in other countries, and only 8% in Benin (Table 4), reflecting the small scale nature of the market vendors in Benin. To illustrate, wholesalers in Benin had a median of 2 staff members [IQR 2–4], while Zambian wholesalers were considerably larger with a median of 8 staff members [IQR 5–15] (Table 4). In Nigeria, Uganda and Zambia, many wholesalers said they were only willing to deliver to customers located nearby.

Wholesalers operating at higher levels of the distribution chain described a range of strategies to reach clients further afield and to increase their market share. In addition to using their own vehicles, wholesalers described engaging couriers and private mass transport companies, such as bus operators in Nigeria and Uganda, to deliver orders to customers. One such method common in Nigeria is called *way billing*, where orders placed by customers are packed by the supplier and transported via mass transit operators (e.g. bus lines) to regional transport hubs, such as bus or taxi parks in commercial centres, from where the customer will retrieve their packaged order.

Sales representatives deployed nationwide to conduct marketing activities and to take and deliver customer orders were commonly used by importers and manufacturers. Vertically integrated supply chains were also encountered in Benin, DRC, Nigeria and Uganda where a drug manufacturer or importer distributed stock from a central warehouse to one or more regional warehouses or wholesale businesses owned by a single enterprise. In Nigeria, a hybrid model was observed, where some domestic manufacturers and importers set up similar distribution nodes by contracting warehousing and distribution services offered by specialised logistics firms.

### Regulation of the Wholesale Pharmaceutical Sector

During the quantitative survey of wholesalers, we documented compliance to a limited number of regulatory requirements that could be easily assessed (Table 5). In all countries, nearly all wholesalers interviewed were observed to store antimalarials appropriately, meaning in dry areas, out of direct sunlight and off the floor. More than 90% of wholesalers in the DRC, Uganda and Zambia also reported employing at least one member of staff with health-related qualifications, most commonly pharmacists, nurses and midwives; compared to Cambodia and Nigeria where two-thirds of wholesalers reported doing so, and only 29% in Benin.

**Table 5:** Regulatory characteristics of wholesalers.

		COUNTRY					
		BENIN	CAMBODIA	DRC	NIGERIA	UGANDA	ZAMBIA
Any up-to-date licence from the pharmaceutical regulatory authority was observed^1^	**%**	**0.9**	**29.5**	**19.9**	**8.7**	**82.0**	**70.0**
	95% CI	0.0*–2.7	20.1–38.8	13.1–26.6	3.9–13.5	75.3–88.8	55.2–84.8
	(N)	(196)	(95)	(136)	(138)	(128)	(40)
An up-to-date wholesale licence from the pharmaceutical regulatory authority was observed^1^	**%**	**0.0**	**10.5**	**15.7**	**8.0**	**63.3**	**55.0**
	95% CI	–	4.2–16.8	9.4–21.9	3.4–12.5	54.8–71.7	38.9–71.1
	(N)	(196)	(95)	(134)	(138)	(128)	(40)
Reported they had been visited by a pharmaceutical inspector in the past year	**%**	**15.2**	**82.4**	**94.2**	**71.2**	**99.2**	**97.5**
	95% CI	8.3–22.0	74.4–90.4	90.3–98.2	63.4–79.0	97.6–100.0*	92.4–100.0*
	(N)	(189)	(91)	(138)	(132)	(123)	(40)
Store antimalarials in a dry area, out of direct sunlight and off the floor	**%**	**91.8**	**88.4**	**90.4**	**97.0**	**92.1**	**86.1**
	95% CI	84.9–98.6	81.5–95.3	84.8–95.9	93.6–100.0*	87.3–96.8	74.2–98.0
	(N)	(154)	(86)	(114)	(100)	(126)	(36)
Employ a member of staff with health qualifications^2^	**%**	**28.7**	**63.4**	**92.8**	**62.8**	**100.0**	**97.6**
	95% CI	18.6–38.8	53.5–73.4	88.4–97.1	54.6–71.0	–	92.8–100.0*
	(N)	(142)	(93)	(138)	(137)	(128)	(42)

CI: confidence interval; N: number of wholesalers contributing to calculation of indicator. 1: DRC pharmaceutical wholesale or retail licences do not possess expiration dates; but these licenses must be maintained on the business premises. 2 Health qualifications common across all countries include pharmacists, pharmacy technicians, pharmacy assistants, nurses, midwives, medical doctors, but some countries may include other categories. Note: 95% confidence intervals are derived using the standard Wald method and confidence limits that have been restricted to the lower limit of 0% and upper limit of 100% are marked with *.

There were also marked differences in the number of wholesalers observed to possess any type of valid licence from the national pharmaceutical regulator, ranging from a high of 82% of wholesalers in Uganda to a low of 1% in Benin. However, in all countries fewer wholesalers were observed to have a valid licence specifically permitting the wholesale of pharmaceuticals. For example, 15% of wholesale businesses in Zambia were operating under a retail or OTC medicines licence; and in Nigeria only 8% of businesses wholesaling antimalarials possessed the required licence to do so, and another 20% reported having only a drug shop/PPMV licence. The large majority of wholesalers in all countries apart from Benin reported that they had been visited by an inspector at least once during the preceding 12 months.

Qualitative investigations revealed a number of common themes around low levels of wholesale licensing compliance. Respondents identified several barriers to obtaining a wholesale pharmaceutical licence, including relatively high administrative fees, difficulties in finding an available and affordable supervising pharmacist, and unclear or overly bureaucratic processes. In several countries, respondents also described corruption as another means to circumvent licensing requirements. For example in the DRC, a few respondents cited instances where unlicensed businesses threatened with forced closure were permitted to continue operating following unofficial payments to regulatory officials. Low levels of enforcement were often attributed to limited resources within national pharmaceutical regulators, where inadequate numbers of inspectors and resources for drug quality testing at points all along the distribution chain restricted the regulator’s capacity to monitor business activity regularly in all parts of the country and continually risked compromising the integrity of the quality assurance chain in the private sector. For example, one large market vendor in Benin cited the ease of accessing comparatively cheaper suppliers in Lagos as the main reason why many vendors in Porto Novo imported illegally. This respondent went on to describe making smaller purchases more frequently when restocking in Nigeria to minimise losses if caught importing illegally; and also transporting stock in a separate vehicle when returning from Lagos to avoid getting apprehended with the illegal goods.

In Cambodia and Nigeria, several respondents suggested that the national regulatory agencies had recently renewed their efforts to improve compliance and reduce the number of unlicensed businesses. Wholesalers in all countries also described their strategies to survive within these complex and sometimes uncertain regulatory environments, such as by building trustworthy supplier relationships to help guarantee product quality and authenticity, or by cooperating with other similar businesses in a number of ways. In Benin, Nigeria, Uganda and Zambia, trade associations organised by activity (e.g. importers, drug shops) or jurisdiction (e.g. specific market or town, national level) provided member benefits such as assistance in achieving and maintaining regulatory compliance; information and training on new regulations, policies and products; access to pooled procurement facilities; and collective representation of members against regulators and policy makers. In Benin and Nigeria, associations of drug vendors operating within traditional markets also performed quasi-regulatory functions by providing members with guidance on the identification and reporting of counterfeit products, and in Benin, conducting surveys of vendors for expired, banned and other substandard products, and imposing penalties on offending businesses.

## Discussion

Our study has produced new nationally representative evidence on the range and extent of interactions among agents working within a pyramidal antimalarial distribution chain, and on the characteristics and practices of the businesses that comprise these chains in each of the study countries. These findings make a substantial contribution to the limited evidence base around distribution chains [4]. In addition to furthering understanding of the complexity of these networks, this new evidence also provides insight into how factors related to regulation, the broader economy and consumer culture shape the market for antimalarial drugs. The study also has important implications for policies and interventions aiming to improve private sector availability, affordability and quality of ACTs [29].

Wholesalers identified consumer demand as the primary determinant of product selection, with the implication that the high prices of ACTs relative to older, less efficacious antimalarials, such as chloroquine and SP, not only impede affordability, but are also a significant barrier to their more widespread availability at both wholesale and retail levels. This highlights the potential for subsidies to increase access to ACT in the private sector, as has been demonstrated by a number of small and large scale subsidy interventions, including the Affordable Medicines Facility – malaria (AMFm) [26], [30]–[33], where subsidies increased ACT market share and improved their availability among retail outlets.

The dominance of consumer demand as a determinant of product selection also suggests that demand shaping activities, such as public awareness and social marketing campaigns, are required to reinforce the effects of price reductions and shift consumer preferences away from more familiar products. This is supported by our observations in Cambodia, the only country in which ACTs dominated wholesaler sales volumes, where ACT subsidies and consumer demand shaping efforts have been most vigorously pursued in response to the development of artemisinin resistance [20]. The need for such supporting interventions for the success of ACT subsidy programmes was also one of the key conclusions from the AMFm evaluation [26].

Minimising the number of distribution chain steps that antimalarials pass through from production to retail level presents an obvious route to reducing consumer prices. At the time of data collection, quality assured ACTs (i.e. products certified by the WHO Prequalification programme) were all imported, unlike more popular nATs of which most were domestically produced. Consequently, it is likely that ACTs pass through more steps than nATs. While building domestic manufacturing capacity for ACTs could help reduce the steps in the supply chain and achieve price reductions, some have argued that building such capacity in malaria endemic countries may not make economic sense because the conditions required to produce high-quality pharmaceuticals are not present, even in countries with otherwise highly developed pharmaceutical sectors [34], [35].

Other findings related to drivers of consumer price and barriers to availability may offer further targets to improve ACT access. For instance, the difficulty and additional costs of transporting goods within countries, particularly in those with large terrains, poor transportation infrastructure and political instability work to limit the geographic penetration and affordability of ACTs. This is particularly the case for quality assured ACTs in those study countries where exclusive distributors tended to be based in urban commercial hubs, making it more difficult for wholesalers in more distant or rural areas to stock ACTs. The range of distribution models used by larger firms in the DRC, Nigeria and Uganda to increase their coverage and market share may present some cost-effective solutions for other settings.

In Benin, Cambodia and the DRC, the limited availability of credit to finance inventory may indicate that wholesalers, particularly those at lower levels of the distribution chain, are constrained from placing larger orders. Because ACTs are relatively higher priced, smaller wholesalers may not be able to order in sufficient quantities to benefit from volume-based discounts or preferred pricing regimes, effectively preventing them from selling ACTs at more competitive prices. Opportunities to pool procurement such as those sometimes facilitated through trade association membership may help to overcome this barrier.

Our findings concerning the low levels of regulatory compliance and the scale of unauthorized antimalarial wholesaling in several countries highlight that efforts to improve end-user antimalarial quality must also account for lapses in quality assurance at wholesale level. At retail level, many countries have taken a more pragmatic approach to reduce the number of unauthorized outlets and improve the quality of pharmaceutical services available to the public by introducing drug shop licenses (e.g. Class C drug shops in Uganda, PPMVs in Nigeria) and accreditation initiatives (e.g. Accredited Drug Dispensing Outlets in Tanzania [36]). In contrast, little has been done to address the issue of unauthorized pharmaceutical wholesaling.

Supporting the formation and activities of trade associations may present a conduit for regulators to engage constructively with unlicensed wholesalers and act to improve practice standards. Reducing some barriers to entering the wholesale market may also help to improve compliance. For example, wholesalers in Benin are required to maintain an operating capital of 100,000,000 CFA (US$ 223,215) [37], which is realistically achievable for only very large firms. Other research suggests that countries with civil (as opposed to common) legal traditions as a post-colonial legacy are associated with heavier barriers to entry and consequently have higher levels of corruption and larger unofficial economies [38]. This may explain some variation in regulatory compliance across study countries (Benin, the DRC and Cambodia have civil legal traditions; Nigeria, Uganda and Zambia have common legal traditions), and understanding the impact of these historical antecedents may also help in the planning of more effective regulatory reforms.

Although this study has broadened knowledge on antimalarial distribution chains and markets, it has also raised additional questions and highlighted priorities for further research. For example, efforts to further improve access to malaria treatment through the private sector will benefit from a better understanding of wholesaler and retailer antimalarial pricing behaviour. Information on different determinants of antimalarial supply and demand could also help to optimise the impact of subsidies and other consumer demand shaping activities. To support rational antimalarial use, it would be useful to investigate how wholesalers could help increase not only the availability of ACTs at retail level, but also the availability of diagnostic testing (i.e. RDTs). Finally, it would also be worthwhile to examine whether our findings related to antimalarials could be generalised to other pharmaceuticals that are easily obtained through the private sector, such as antibiotics.

A key limitation of the study relates to the potential sensitivity of some of the topics, which might contribute to social desirability bias, with respondents’ answers reflecting what they believe the interviewer would find acceptable. Also, data from qualitative interviews were documented using a note taker, rather than being recorded. While this may have helped to improve the validity of the data by allowing respondents to be more at ease, some of the richness and detail of the discourse is likely to have been lost. Missing supplier information from retail outlets may have also biased wholesaler sampling frames toward more registered types of suppliers; however, our innovative ‘bottom-up’ sampling approach did identify considerable numbers of unregistered wholesalers in all countries that were included in our samples. Finally, data for this study were collected in 2009–2010 and changes to the market since then are likely to have occurred, particularly following the piloting of the AMFm in Nigeria and Uganda in 2011–2012.

## Supporting Information

Text S1 **Details on sampling in markets and the calculation of weighted summary measures in Benin.**
(DOC)

Text S2 **Additional details on estimating weekly antimalarial sales volumes.**
(DOC)
