# Supplementary material for: Understanding Private Sector Antimalarial Distribution Chains: A Cross-Sectional Mixed Methods Study in Six Malaria-Endemic Countries
Source: PLoS One. 2014 Apr 3;9(4):e93763. doi: 10.1371/journal.pone.0093763 (PMC3974780; doi:10.1371/journal.pone.0093763)
Supplement: Text S1 — Details on sampling in markets and the calculation of weighted summary measures in Benin. (DOC) [file pone.0093763.s001.doc]

**Text S1: Details on sampling in markets and the calculation of weighted summary measures in Benin**

In Benin, traditional markets that are common throughout West Africa are important sources of antimalarial wholesaling. Many of the businesses selling antimalarials in these markets tend to be concentrated in Cotonou and Porto-Novo, but also in a number of smaller regional towns. When retailers and wholesalers in Benin were asked about their top two antimalarial supply sources, respondents commonly indicated that they purchased antimalarials from such markets, and often did not name a specific vendor in the market as their supply source, giving just the market name as the source of antimalarials. In a minority of instances, respondents would mention the name of the market vendor, and in some of these cases, data collectors were able to identify the specific vendor.

For the mentions within markets where a specific business was not named, it was difficult to ascertain which and how many wholesalers needed to be interviewed in a given market. In addition, some potential wholesaler respondents were reluctant to participate in the study due to political and regulatory sensitivities surrounding medicine-selling in markets. To address these issues, attempts were made to sample as wide a selection of wholesalers that was possible within each market visited, often with the assistance of local leaders or PSI/Benin staff responsible for working with market wholesalers who accompanied data collection teams to try to foster goodwill in the marketplace and encourage participation among vendors. Also, because markets do not all operate every day of the week, it was not possible to survey market wholesalers in each of the market towns mentioned, in which case wholesalers were sampled from markets in nearby towns to represent them. Ideally, the number of wholesalers interviewed in a particular market would be proportional to the number of mentions each market received; however, for the reasons mentioned above, this was not always the case. Therefore, summary measures and their confidence intervals that include observations from markets were adjusted to account for over- or under-sampling of market wholesalers that may have occurred.

To assess whether over- or under-sampling of market wholesalers had occurred, data on sources of antimalarial supply (i.e. two top antimalarial wholesalers) collected both from antimalarial retailers and wholesalers were used to calculate the frequency with which market versus non-market wholesalers were mentioned, and the frequency with which different markets were mentioned. These data on number of supplier mentions were used to calculate pseudo-probability analytical weights that adjusted summary measures presented in this report to account for the over- or under-sampling of wholesalers in markets. Weights were derived using the following formula:

$$W_{M_{i}}=\frac{I_{M_{i}}^{'}}{I_{M_{i}}}=\frac{[M_{M_{i}}/(M_{NM}+\sum M_{M_{i}})]\cdot[I_{NM}\cdot(M_{NM}+\sum M_{M_{i}})/M_{NM}]}{I_{M_{i}}}=\frac{M_{M_{i}}\cdot I_{NM}}{M_{NM}\cdot I_{M_{i}}}$$

where:

$W_{M_{i}}$ is the market-specific analytical weight

$I_{M_{i}}^{'}$ is the number of interviews that should have been conducted among wholesalers based in market *i*

$I_{M_{i}}$ is the total number of interviews conducted among wholesalers based in market *i*

$I_{NM}$ is the total number of interviews conducted among non-market-based wholesalers

$M_{M_{i}}$ is the total number of mentions for wholesalers in market *i*

$M_{NM}$ is the total number of mentions for non-market-based wholesalers

The general approach to calculating and applying weights was as follows:

1. Using the total combined list of supplier mentions gathered from both retailers and wholesalers, proportions of specific non-market and all market supplier mentions were calculated. Among market supplier mentions, similar proportions were calculated for individual markets by dividing the number of mentions for a particular market by the total number of supplier mentions (market and non-market). Supplier mentions from respondents that did not provide sufficient information to identify a business or market were removed from the total list of supplier mentions for the calculation of weights as it was impossible to classify these mentions as either market or non-market wholesalers. At retail level, many of these were ambulatory antimalarial vendors; as these were so difficult to identify, this group of wholesalers is likely to be under-represented in the data presented.
2. In the absence of a reliable sampling frame, we assumed that this relative frequency distribution of mentions approximated the actual distribution of wholesalers in Benin, and used it post hoc as the basis to calculate the number of market-based wholesalers that should have been interviewed in each market, and subsequently to derive the analytical weights to adjust for over- and under-sampling across different markets.
3. For non-market wholesalers, individual weights were always set to 1 as it was assumed that no over- or under-sampling took place for non-market suppliers. However, it is possible that some under-sampling of non-market suppliers did take place where it was not possible to identify mentioned wholesalers; but, this was relatively rare.
4. Using the total number of non-market wholesalers actually interviewed as a constant and the frequency distribution of total mentions, the total number of all wholesalers that should have been interviewed was calculated by dividing the number of non-market wholesalers actually interviewed by the proportion that non-market wholesalers represented in the overall frequency distribution of all mentions.
5. The number of wholesalers that should have been interviewed in each market according to the frequency distribution of mentions was then determined by multiplying the total number of all wholesalers that should have been interviewed by the proportion of mentions for a given market relative to the total number of all mentions.
6. Market-specific weights were calculated for wholesalers operating in these markets as the number of wholesalers that should have been interviewed according to the frequency distribution of mentions divided by the actual number of market wholesalers interviewed. In one market where wholesalers were under-sampled, this produced a weight greater than 1 to ensure greater representation of these particular observations in the calculation of summary measures; weights were less than 1 where oversampling of market wholesalers occurred.

Depending on the Stata command, weights were applied using the *aweight* options to calculate medians with their inter-quartile range, or using the *pweight* option to calculate proportions with their 95% confidence intervals.

While markets were also commonly mentioned as sources of antimalarial wholesale suppliers in Nigeria, specific businesses were named in nearly all mentions; unnamed market wholesalers were not mentioned by other wholesalers, and only mentioned by a small minority of retailers. As such, weights were not used in the calculation of summary measures in Nigeria. Apart from Benin and Nigeria, traditional markets did not appear to be important sources of antimalarial wholesaling in the remaining study countries, so weights were not used in the calculation of summary measures.
