# Supplementary material for: Understanding Private Sector Antimalarial Distribution Chains: A Cross-Sectional Mixed Methods Study in Six Malaria-Endemic Countries
Source: PLoS One. 2014 Apr 3;9(4):e93763. doi: 10.1371/journal.pone.0093763 (PMC3974780; doi:10.1371/journal.pone.0093763)
Supplement: Text S2 — Additional details on estimating weekly antimalarial sales volumes. (DOC) [file pone.0093763.s002.doc]

**Text S2: Additional details on estimating weekly antimalarial sales volumes**

Antimalarial volumes were calculated on the basis of an adult equivalent treatment dose (AETD). An AETD was defined as the number of milligrams (mg) of an antimalarial drug needed to treat a 60 kg adult. The number of mg/kg used to calculate one AETD was defined as what was, at the time of the study, recommended for a particular drug combination in the treatment guidelines for uncomplicated malaria in areas of low drug resistance issued by the World Health Organization. Where these treatment guidelines did not exist, AETDs were based on product manufacturers’ treatment guidelines. In the case of ACTs as the treatment consists of 2 or more active antimalarial ingredients packaged together (either co-formulated or co-blistered), the strength of the artemisinin-based component was used as the principal ingredient for the AETD calculations. Information collected on both the medicine strength and unit size, as listed on the product packaging, was then used to calculate the number of AETDs contained in each unit. The median number of antimalarial doses reported to have been sold during the week preceding the survey was estimated for each antimalarial category for all wholesalers by first summing the number of AETDs sold for the different antimalarial categories at each wholesale outlet and then by taking the median across all wholesalers.

For wholesalers that stocked antimalarials and for which some or all sales volumes were missing, missing values were imputed using the Stata 11 command *mi impute pmm* (an approach for imputing missing values of one continuous variable whose distribution is skewed). Missing values (e.g. in the case of a wholesaler stocking antimalarials and with the antimalarial type identified in the inventory sheet but for which sales volume data were missing) were imputed using covariates related to wholesaler and product characteristics. Five imputations were conducted and their mean imputed to the missing values. For eligible wholesale outlets with no antimalarials of a given category in stock at the time of the survey, sales volumes over the past week were assumed to be null. For wholesale outlets without information about the type of antimalarials stocked (because of refusals to participate in the study or to provide information on the type of antimalarials stocked or because of interrupted interviews), sales volumes were treated as missing.
